# Trimeric complexes of Antp-TBP with TFIIEβ or Exd modulate transcriptional activity

**DOI:** 10.1186/s41065-022-00239-8

**Published:** 2022-05-30

**Authors:** Gustavo Jiménez-Mejía, Rubén Montalvo-Méndez, Carolina Hernández-Bautista, Claudia Altamirano-Torres, Martha Vázquez, Mario Zurita, Diana Reséndez-Pérez

**Affiliations:** 1 Universidad Autónoma de Nuevo León, Facultad de Ciencias Biológicas, Departamento de Inmunología y Virología, San Nicolás de los Garza, Nuevo León, México; 2 Universidad Autónoma de Nuevo León, Facultad de Ciencias Biológicas, Departamento de Biología Celular y Genética, San Nicolás de los Garza, Nuevo León, México; 3 Universidad Nacional Autónoma de México, Instituto de Biotecnología, Departamento de Fisiología Molecular y Genética del Desarrollo, Cuernavaca, Morelos, México

**Keywords:** Antp, TBP, TFIIEβ, Exd, Trimeric complexes, BiFC-FRET

## Abstract

**Background:**

Hox proteins finely coordinate antero-posterior axis during embryonic development and through their action specific target genes are expressed at the right time and space to determine the embryo body plan. As master transcriptional regulators, Hox proteins recognize DNA through the homeodomain (HD) and interact with a multitude of proteins, including general transcription factors and other cofactors. HD binding specificity increases by protein–protein interactions with a diversity of cofactors that outline the Hox interactome and determine the transcriptional landscape of the selected target genes. All these interactions clearly demonstrate Hox-driven transcriptional regulation, but its precise mechanism remains to be elucidated.

**Results:**

Here we report Antennapedia (Antp) Hox protein–protein interaction with the TATA-binding protein (TBP) and the formation of novel trimeric complexes with TFIIEβ and Extradenticle (Exd), as well as its participation in transcriptional regulation. Using Bimolecular Fluorescence Complementation (BiFC), we detected the interaction of Antp-TBP and, in combination with Förster Resonance Energy Transfer (BiFC-FRET), the formation of the trimeric complex with TFIIEβ and Exd in living cells. Mutational analysis showed that Antp interacts with TBP through their N-terminal polyglutamine-stretches. The trimeric complexes of Antp-TBP with TFIIEβ and Exd were validated using different Antp mutations to disrupt the trimeric complexes. Interestingly, the trimeric complex Antp-TBP-TFIIEβ significantly increased the transcriptional activity of Antp, whereas Exd diminished its transactivation.

**Conclusions:**

Our findings provide important insights into the Antp interactome with the direct interaction of Antp with TBP and the two new trimeric complexes with TFIIEβ and Exd. These novel interactions open the possibility to analyze promoter function and gene expression to measure transcription factor binding dynamics at target sites throughout the genome.

**Supplementary Information:**

The online version contains supplementary material available at 10.1186/s41065-022-00239-8.

## Introduction

Hox proteins are transcription factors (TFs) that coordinate antero-posterior morphogenesis during embryo development [1–5]. They are characterized by a highly conserved DNA-binding homeodomain (HD) that recognizes small, highly frequent DNA sequences [6]. Although HDs are highly similar in structure and affinity, they regulate targets in a very specific space- and time-dependent manner, which raises the question of how they can recognize similar DNA sequences with high affinity to modulate target genes for functional specificity, leading to the so-called “Hox paradox”. It has been reported that protein–protein interactions are crucial for the specificity of homeoproteins including cell–matrix proteins, chromatin remodeling complexes, cofactors and even non-coding RNAs [7–11]. Interactions with cofactors such as Extradenticle (Exd) and other transcriptional factors through the Hox proteins short linear motifs like the YPWM or UbdA are essential for Hox activity [12–14].

Of particular interest are the protein interactions that occur between Hox proteins and general transcription factors (GTF) from the RNA Pol II basal transcription machinery. Homeoproteins like Msx-1, Even- skipped (Eve), Pax5 and Pax6 also interact with TATA-binding protein (TBP) for transcriptional regulation [15–18]. Several homeoproteins interact with the Med19 subunit of MED complex, TFIIEβ and M1BP, a pausing Pol II factor involved in chromatin remodeling [14, 19, 20]. Antennapedia (Antp) interacts with the Bric-a-brac interacting protein (BIP2/TAF3) through the YPWM motif [21] and with TFIIEβ, specifically through the 32 and 36 positions of HD helix 2 [22]. Additionally, *Drosophila* and mouse Hox proteins form trimeric complexes with Exd-Homothorax (Hth) and MEIS-PBX respectively [23, 24]. This plethora of Hox protein–protein interactions clearly points to a Hox-driven transcription process in which every Hox homeoprotein could selectively recruit GTFs to achieve specificity and activate or repress target genes during *Drosophila* development, although the precise molecular mechanisms remain elusive [25].

Here, we focused on the interplay between Antp and the basal transcription machinery and show the direct interaction of Antp with TBP through the poly-glutamine (PolyQ) regions of both proteins. Furthermore, we found new trimeric complexes between Antp-TBP and TFIIEβ or Exd, which modulate Antp transcriptional activity. Our results provide important insights into the molecular mechanisms of the Antp interactome with the basal transcription machinery and contribute to the intriguing molecular mechanisms by which the Hox interactome drives transcriptional regulation.

## Results

### Antp directly interacts with TBP through its N-terminal region

**Fig. 1:**
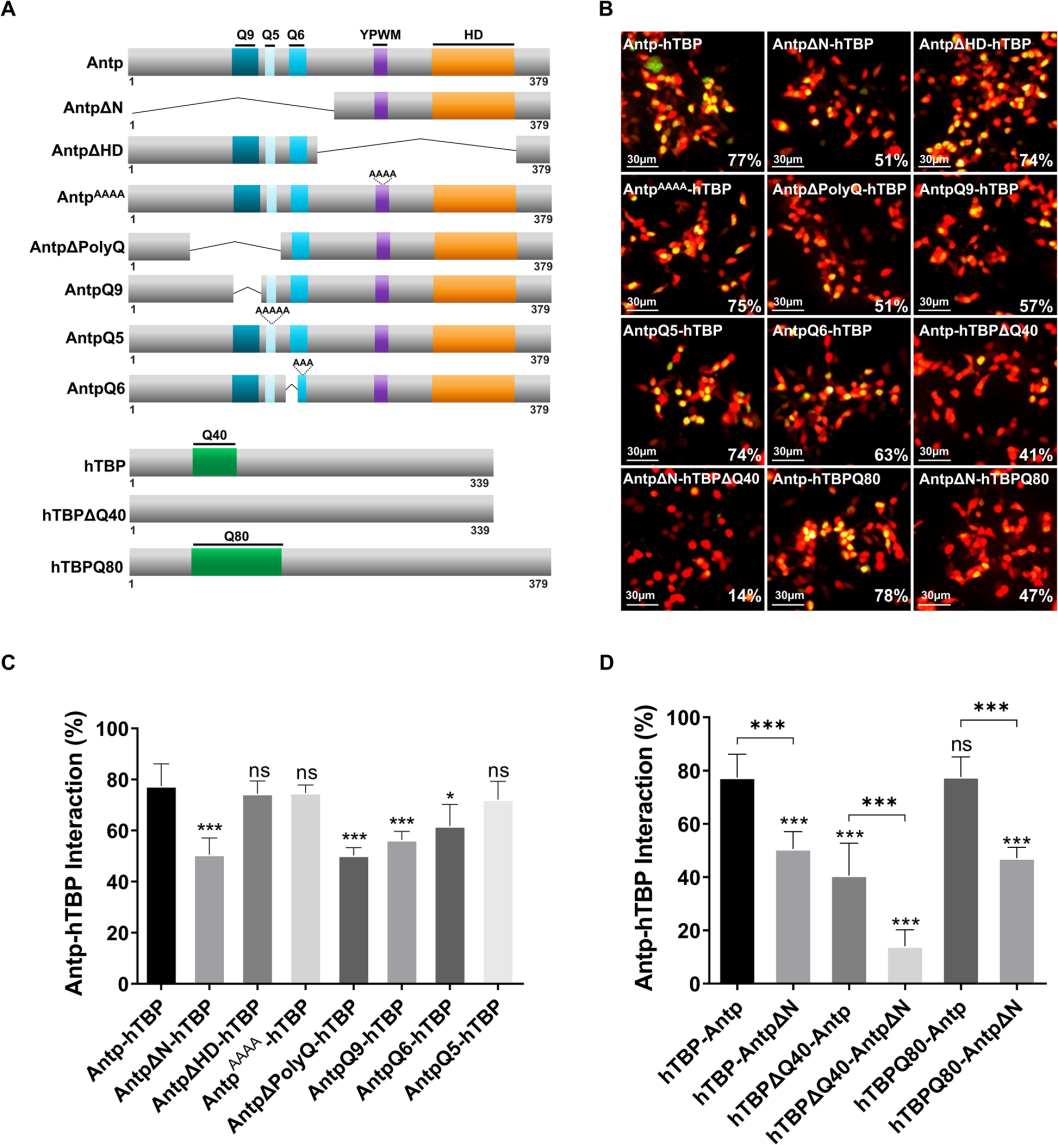
Antp and hTBP interact through polyglutamine stretches. Antp-hTBP interaction was determined using BiFC assays in transfected HEK293 cells. **A** Schematic representations of Antp and hTBP with their mutant versions. For Antp, the polyQ regions are indicated in shades of blue; YPWM, in purple; and the homeodomain (HD), in orange. For hTBP, the polyQ region is indicated in green. **B** BiFC assays revealed an interaction between Antp and hTBP (Venus reconstitution in green) and a reduction of BiFC between Antp and hTBP polyQ mutants. The number on the lower right corner indicates the percentage of cells showing the interaction. **C** Statistical analysis of BiFC-detected interactions between Antp-mutant versions and hTBP. AntpΔHD/^AAAA^/Q5-hTBP showed no significant difference, in contrast, there is a highly significant difference (***) with AntpΔN/ΔPolyQ/Q9-hTBP. AntpQ6-hTBP interaction showed a significant difference (*). **D** Statistical analysis of BiFC-detected interactions between wild-type and mutant Antp-hTBP versions. hTBPΔQ40-Antp interaction showed a highly significant difference (***) in contrast with a non-significant difference for hTBPQ80-Antp. Antp or ΔN interactions with hTBP or its mutant versions have a highly significant difference in all pairs compared. pCAG-mCherry (red fluorescence) was used as an internal control. Three independent triplicates were analyzed using one-way ANOVA and the post-hoc test Tukey for mean comparison; all significant differences were obtained comparing to Antp-hTBP interaction. Significance is indicated as *** = *p* ≤ 0.001, ** = *p* ≤ 0.01, * = *p* ≤ 0.05, ns is not significant, and Error bars correspond to standard deviation. Scale bar, 30 µm

To determine the interaction between Antp and the basal transcription factor TBP, we performed Bimolecular Fluorescence Complementation (BiFC) assays in human HEK293 cells. Our results showed the interaction between Antp and TBP on 77% of transfected cells (Fig. 1B). In order to characterize the Antp-TBP protein–protein interaction, we carried out a series of deletions and site-directed mutagenesis on both proteins (Fig. 1A, Fig. S1 and S2). The absence of Antp N-terminal (AntpΔN) decreased the interaction with TBP to 51% with a highly significant difference compared to 77% of Antp and 74% of the YPWM-HD deletion in AntpΔHD. We also analyzed the interaction of the Antp YPWM motif substitution with alanines (Antp^AAAA^), which also showed no significant effect in the interaction with TBP (Fig. 1B and C). These results clearly indicate that the N-terminal of Antp is important for its interaction with TBP, and neither the HD nor the YPWM motif is required for this interaction in cell culture.

### Polyglutamine stretches of Antp and TBP are required for interaction

Since the Antp N-terminal is a PolyQ-rich region important for the interaction with TBP, we performed PolyQ stretch deletions (AntpΔPolyQ and AntpQ9) or alanine substitutions (AntpQ6 and AntpQ5) on the Antp N-terminal (Fig. 1A and S1). A highly significant reduction to 57% with AntpQ9 and 51% with AntpΔPolyQ was found (Fig. 1B and C). The simultaneous deletion and substitution of the AntpQ6 mutant version also significantly reduced the interaction to 63%, whereas mutagenesis of the AntpQ5 stretch maintained the interaction with TBP with non-significant difference (74%), compared to the wild-type Antp-TBP interaction (77%) (Fig. 1B and C). Our results indicated that Q9 and Q6 PolyQ stretches directly participate in the interaction with TBP and that Q5 stretch is not involved in the interaction.

Given the importance of Antp PolyQ regions, we wondered whether TBP PolyQ is also involved in the interaction. Deletion of the TBP PolyQ (TBPΔQ40) reduced its interaction with Antp to 41% with a highly significant difference (Fig. 1A, B, D and S2). Accordingly, the absence of both PolyQ regions from Antp (AntpΔN) and TBP (TBPΔQ40) caused a highly significant reduction to 14% (Fig. 1B and D). Additionally, an expanded TBP homopeptide (TBPQ80) did not affect the interaction, showing no significant difference (78%) compared to the wild-type Antp-TBP interaction (77%). In contrast, in absence of Antp PolyQs (AntpΔN), the interaction with TBPQ80 diminished to 47% (Fig. 1B, D and S2). Altogether, these results indicate that PolyQ regions in both Antp and TBP are important for the interaction, and a longer TBPQ80 has no effect on it.

### Trimeric complex formation of Antp-TBP with TFIIEβ or Exd

To determine whether Antp-TBP could form trimeric complexes with other TFs, we first standardized a BiFC-FRET combination approach in living cells, using the Jun-Fos-p65 trimer (Fig. S3) as previously reported [26].

**Fig. 2:**
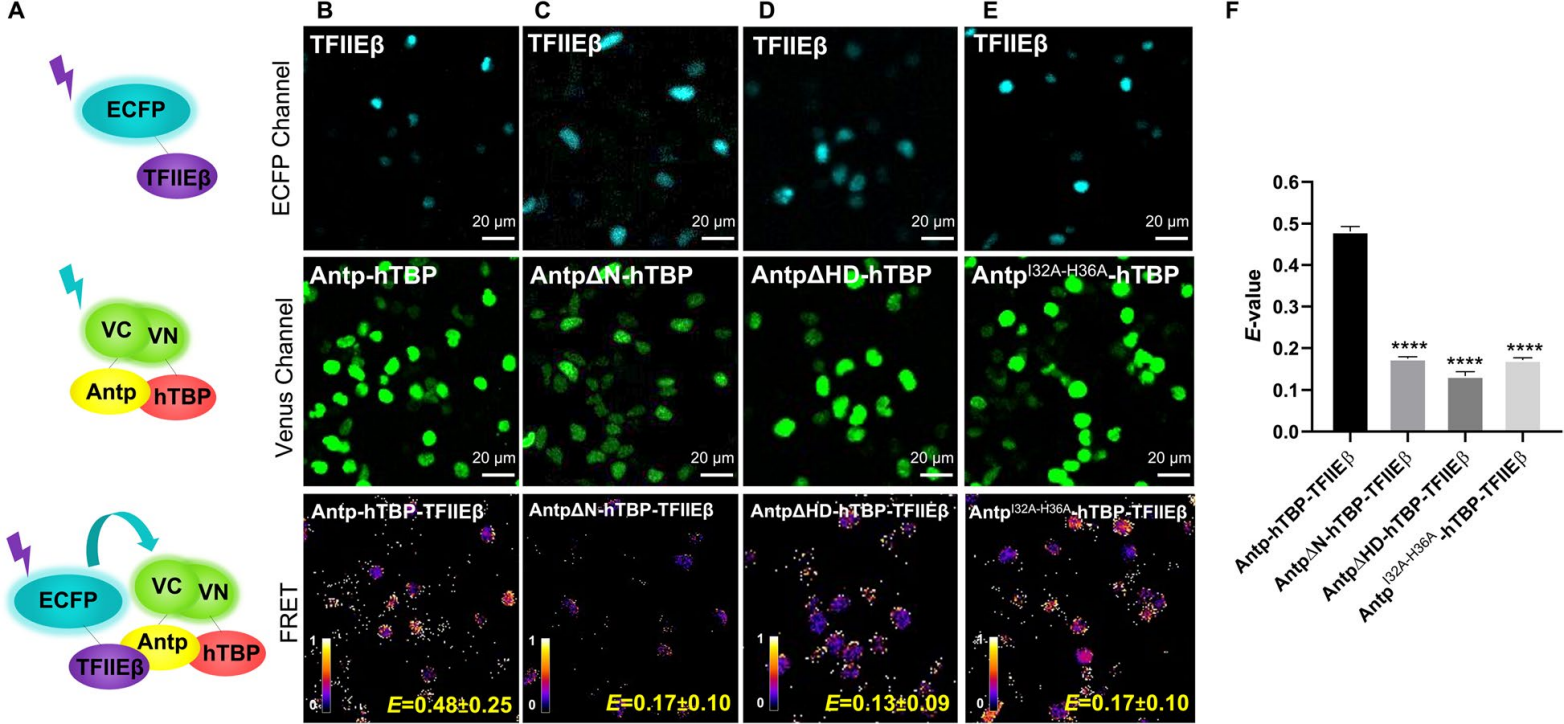
Trimeric complex formation of Antp-hTBP with TFIIEβ by BiFC-FRET. **A** Schematic representation of TFIIEβ fused to cyan (ECFP Channel), BiFC by Antp-hTBP interaction (Venus channel), and energy transfer due to Antp-hTBP-TFIIEβ trimeric complex (FRET). **B** TFIIEβ trimeric interaction with Antp-hTBP heterodimer (*E* = 0.48 ± 0.25). **C** AntpΔN diminishes trimeric complex formation (*E* = 0.17 ± 0.10). **D** HD deletion of Antp (AntpΔHD) decreases the trimeric complex formation (*E* = 0.13 ± 0.09). **E** The Antp helix 2 residues 32 and 36 are important for the trimeric complex (*E* = 0.17 ± 0.10). Color bar represents FRET intensity (Fire mode); darker color indicates low trimeric interaction levels and lighter color indicates high trimeric interaction levels. **F** The graph shows statistical analysis of three independent triplicates using a one-way ANOVA for mean comparison, significance is indicated as *** = *p* ≤ 0.001, ** = *p* ≤ 0.01, * = *p* ≤ 0.05 and error bars correspond to standard error (*p* ≤ 0.001). Scale bar, 20 µm

**Fig. 3:**
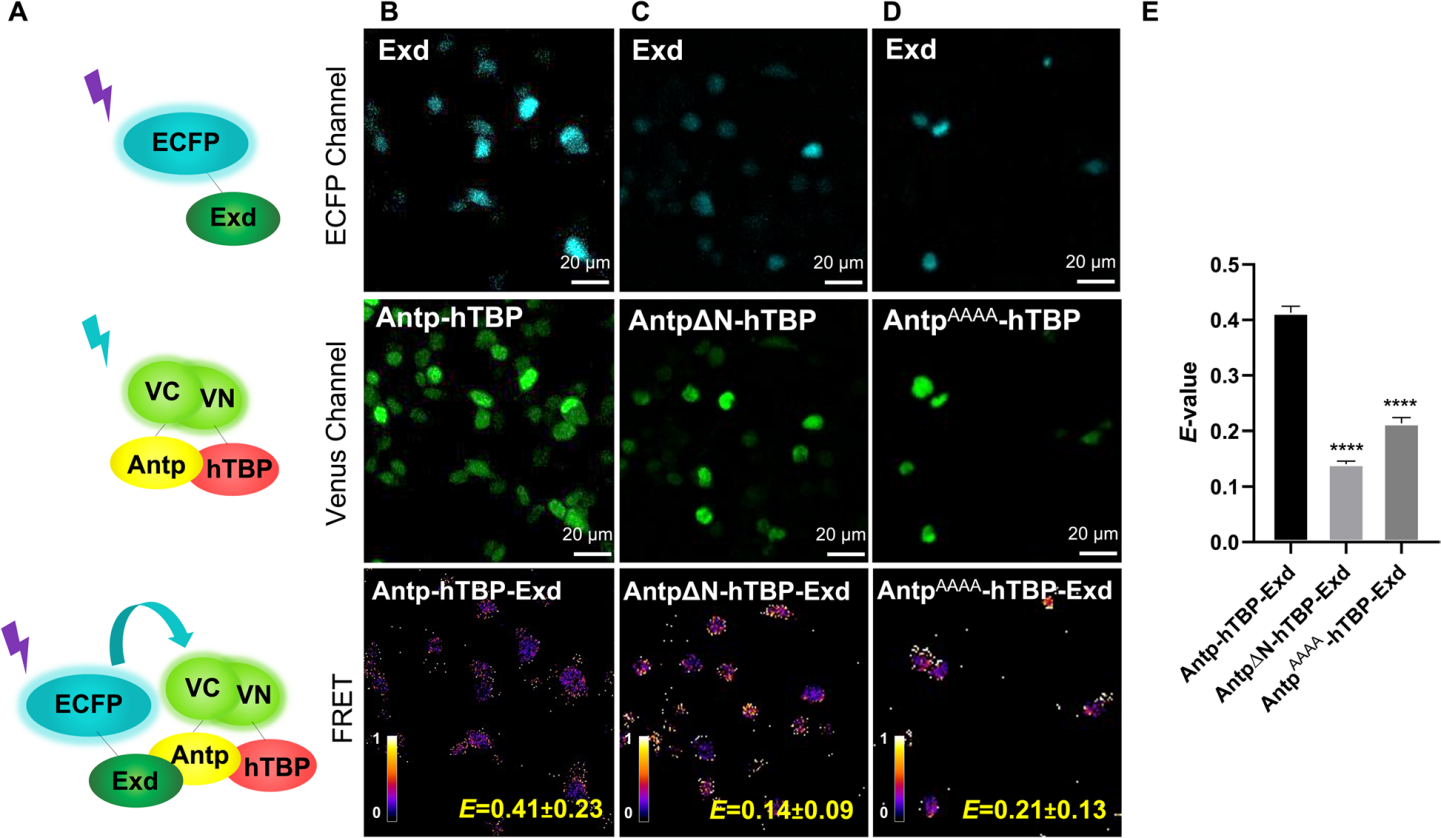
Exd forms a trimeric complex with Antp-hTBP by BiFC-FRET. **A** Schematic representation of Exd fused to cyan (ECFP Channel), BiFC by Antp-hTBP interaction (Venus channel), and energy transfer due to Antp-hTBP-Exd trimeric complex (FRET). **B** Exd trimeric interaction with Antp-hTBP heterodimer (*E* = 0.41 ± 0.23). **C** Antp-ΔN diminished trimeric complex formation (*E* = 0.14 ± 0.09). **D** Antp YPWM mutant (Antp^AAAA^) affects trimeric interaction (*E* = 0.21 ± 0.13). Color bar represents FRET intensity (Fire mode); darker color indicates low trimeric interaction levels and lighter color indicates high trimeric interaction levels. **E** The graph shows statistical analysis of three independent triplicates using a one-way ANOVA for mean comparison, significance is indicated as *** = *p* ≤ 0.001, ** = *p* ≤ 0.01, * = *p* ≤ 0.05 and error bars correspond to standard error (*p* ≤ 0.001). Scale bar, 20 µm

**Fig. 4:**
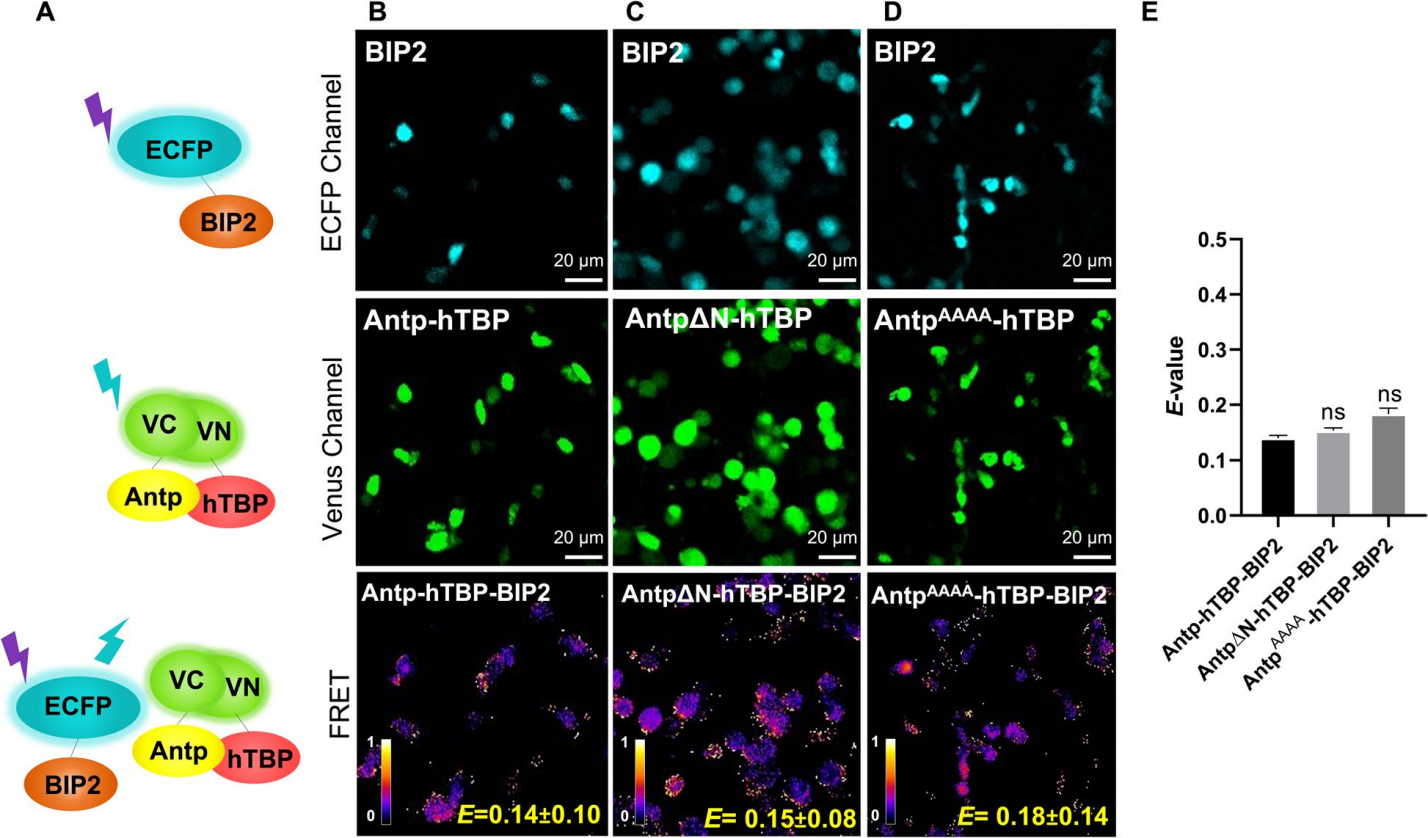
BIP2 does not form a trimeric complex with Antp-hTBP by BIFC-FRET. **A** Schematic representation of BIP2 fused to cyan (ECFP Channel), BiFC by Antp-hTBP interaction (Venus channel), and lack of energy transfer between BIP2 and Antp-hTBP heterodimer (FRET panel). **B** BIP2 does not form trimers with Antp-hTBP heterodimer (*E* = 0.14 ± 0.10). **C** AntpΔN does not modify trimeric complex formation (*E* = 0.15 ± 0.08). **D** Antp YPWM mutant (Antp^AAAA^) does not modify trimeric interaction (*E* = 0.18 ± 0.14). Color bar represents FRET intensity (Fire mode); darker color indicates low trimeric interaction levels and lighter color indicates high trimeric interaction levels. **E** The graph shows statistical analysis of three independent triplicates by one-way ANOVA for mean comparison. Error bars correspond to standard error (*p* ≤ 0.001), ns is not significant. Scale bar, 20 µm

We used TFIIEβ, Exd and BIP2 fused to ECFP as donors and VCAntp-VNTBP interaction (Venus reconstitution) as acceptor (Fig. 2A, 3A and 4A). Formation of trimeric complexes between Antp-TBP and TFIIEβ was shown clearly with a high *E value* (0.48 ± 0.25) (Fig. 2B, lower panel). Disruption of Antp-TBP interaction by the AntpΔN mutation caused a highly significant reduction of the trimeric complex (*E* = 0.17 ± 0.10; Fig. 2C and F). In the same way, either the HD deletion of Antp in AntpΔHD or the HD helix 2 mutant Antp^I32A−H36A^ [22] reduced the formation of the Antp-TBP-TFIIEβ trimer in a highly significant manner (*E* = 0.13 ± 0.09 and *E* = 0.17 ± 0.10 respectively; Fig. 2D-F). Together, these results validated the novel formation of the trimeric complex Antp-TBP-TFIIEβ, because the trimer is not formed when the domains involved in the dimeric interactions are missing in the Antp mutants.

Next, we found the formation of Antp-TBP-Exd trimer (*E* = 0.41 ± 0.23; Fig. 3A and B, lower panel). This trimeric interaction was corroborated by disruption of the Antp-TBP dimer using the AntpΔN mutant, which showed a highly significant reduction of the trimeric complex (*E* = 0.14 ± 0.09; Fig. 3C and E). Similarly, the Antp YPWM mutant (Antp^AAAA^) decreased the formation of the trimeric complex in a highly significant manner (*E* = 0.21 ± 0.13; Fig. 3D and E). Our results clearly corroborated the Antp-TBP-Exd trimer formation.

By contrast, we did not find the trimer formation between BIP2 and Antp-TBP (*E* = 0.14 ± 0.10; Fig. 4A and B). Accordingly, both AntpΔN and Antp^AAAA^ mutants used to disrupt dimeric interactions showed no significant difference (*E* = 0.15 ± 0.08 and *E* = 0.18 ± 0.14 respectively; Fig. 4C-E). These results corroborated that BIP2 does not form a trimeric complex with Antp-TBP.

### Transcriptional function of Antp complexes with TBP, TFIIEβ or Exd

**Fig. 5:**
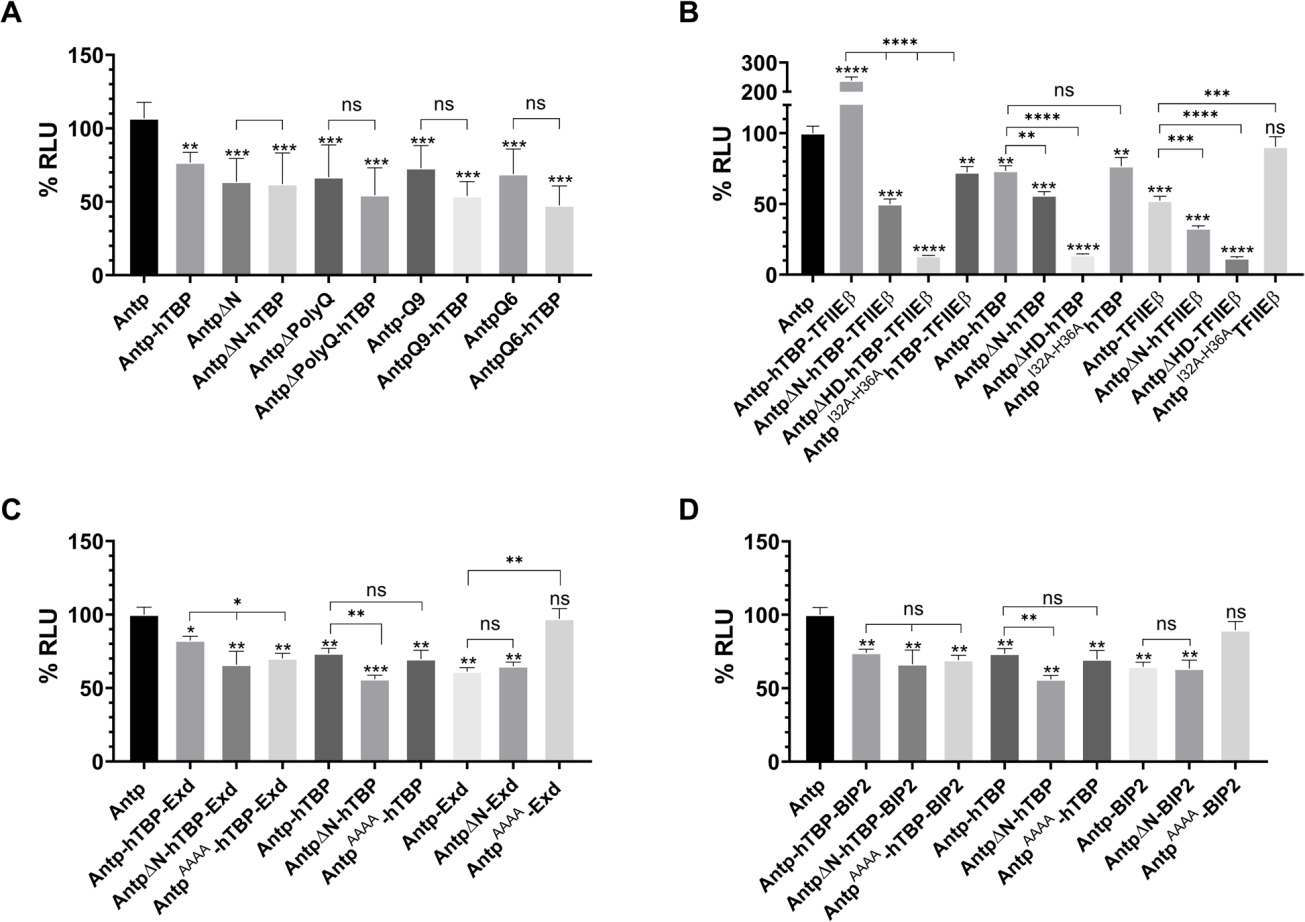
Antp complexes modulate their transcriptional activity. **A** The graphic shows the percentage of transcriptional activation mediated by Antp and its mutant versions in the presence or absence of hTBP. **B** Transcriptional activity of Antp compared with the one obtained with co-expression of trimeric Antp-hTBP-TFIIEβ complexes, Antp-hTBP, and Antp-TFIIEβ dimeric complexes. **C** Transactivation effect of Antp-hTBP-Exd trimeric complex, Antp-hTBP, and Antp-Exd dimers. **D** Transcriptional activity of Antp-hTBP-BIP2, Antp-hTBP, and Antp-BIP2 interactions. Statistical analysis of three independent triplicates was made using a one-way ANOVA and the post-hoc test Tukey for mean comparison. Error bars correspond to standard deviation (*p* < 0.005)

We next determined the effect of the complexes on Antp transcriptional activity using a luciferase (LUC) reporter (pGLH11) with a minimal Hsp70 promoter and eleven BS2 binding sites recognized by Antp helix 3 (Fig. S4). The relevance of PolyQ regions was confirmed with the Antp mutants ΔN, ΔPolyQ, Q9, and Q6 that reduced significantly Antp transactivation activity. Antp co-expressed with TBP had a statistically significant reduction to 76% of LUC expression compared to Antp, indicating that its transcriptional activity is affected by the presence of TBP. Moreover, there is no significant difference in the transcriptional activity between the mutant versions AntpΔN, ΔPolyQ, Q9, and Q6 compared with their co-expression with TBP (Fig. 5A).

Interestingly, the Antp-TBP-TFIIEβ trimer shows a highly significant increase to 238% in Antp transcriptional activity (Fig. 5B). Trimer disruption with Antp mutants drastically decreases trimer transactivation levels to 49% (ΔN), 13% (ΔHD) and 72% (Antp^I32A−H36A^). Antp transcriptional activity decreased to 73% for Antp-TBP and 52% for Antp-TFIIEβ (Fig. 5B). Antp mutants with TBP or TFIIEβ decreased transcriptional activity: AntpΔN-TBP to 56%, and AntpΔHD-TBP to 14%. In the same manner, AntpΔN-TFIIEβ was also reduced to 52%, and AntpΔHD-TFIIEβ to 32%. As expected, Antp^I32A−H36A^ with TFIIEβ shows levels of transactivation very similar to Antp (90%). Our results clearly indicated that Antp-TBP-TFIIEβ trimer significantly increased transcriptional activity whereas the dimers of Antp with TBP or TFIIEβ diminish it. Altogether, these results support that the formation of trimer Antp-TBP-TFIIEβ enhances Antp transcriptional activation.

We also found that Antp-TBP-Exd complex reduced significantly Antp transcriptional activity to 82% (Fig. 5C). The disruption of the trimer caused a significant reduction of transactivation to 65% (ΔN) and 70% (Antp^AAAA^). Antp dimers also decrease activity to 73% with TBP and 61% with Exd. The use of AntpΔN mutant to interfere with the formation of Antp-TBP showed a decrease of the transcriptional activity and does not have a significant effect on Antp-Exd as expected. Likewise, Antp^AAAA^ mutant with Exd has a transactivation level of 97%, very similar to Antp (Fig. 5C). These indicate that both trimeric and dimeric complexes between Antp, TBP and Exd reduced Antp transcriptional activity.

Furthermore, co-expression of Antp, TBP, and BIP2 reduced Antp transactivation to 74%, and the use of the mutant ΔN or Antp^AAAA^ did not show any significant difference (Fig. 5D), supporting the results described above for the lack of assembly of the trimeric complex. It is important to indicate that dimers showed the same transactivation activity. As expected, disrupting Antp-BIP2 dimer with Antp^AAAA^ mutant recovered Antp transactivation (Fig. 5D), indicating the YPWM effect on the dimer formation.

## Discussion

Here, we increased the repertoire of Hox interacting partners by identifying the interaction of Antp with TBP and the formation of novel trimeric complexes with TFIIEβ and Exd, but not with BIP2 in living cells. Antp-TBP interaction involves both PolyQ regions, and they are also important for Antp transactivation activity. Furthermore, the trimeric complexes with TFIIEβ and Exd modulate transcriptional regulation.

Antp-TBP interaction is relevant because TBP plays a central role in transcriptional regulation as a target for distinct activator and repressor proteins [27–33] and homeoproteins [15–18]. TBP interacts with CDX1 via the HD but not with CDX2, supporting the selective interaction of TBP with homeoproteins [34]. Like other homeoproteins, Antp interacts with other members of the basal transcription machinery as BIP2/TAF3, Med19, TFIIEβ and M1BP [14, 19–22], denoting the implication of homeoproteins in the Preinitiation Complex (PIC).

The Antp PolyQ region is responsible for TBP interaction, as previously reported in pull-down assays [35], and this interaction is directly related to the number of glutamines, supporting the function of the 9- and 6-PolyQ stretches. Previous studies point out that PolyQ stretches as protein–protein interaction motifs generally are related with transcriptional regulation [36, 37]. We also found that the PolyQ stretch of TBP is relevant for Antp interaction because its absence diminishes BiFC. This decrease is in accordance to a previous report of C-terminal domain of TBP involvement in Antp interaction [35]. Preliminary results of BiFC assays in *Drosophila melanogaster* TBP (dmTBP) showed similar percentage of interaction with Antp in a PolyQ-dependent manner, which is also supported by previous in vitro experiments due to the high structural similarity between hTBP and dmTBP [35, 38]. Antp-TBP interaction decreased even more in absence of both proteins’ PolyQ regions. Similarly, it was previously reported that Ataxin 7 (SCA7) and the homeobox protein Crx interact through the PolyQ stretches from both proteins and this interaction can regulate transcriptional activity [39].

Our results show that Antp without its N-terminal region (AntpΔN) reduced its transactivation activity in the same way as previously reported with HoxA5 [40], indicating that PolyQ regions enhance transactivation activity, even though the HD is necessary for DNA-binding [6, 41]. These results are in concordance with previous reports indicating that the PolyQ region of Antp and Sp1 are required to activate transcription [42, 43].

Our results show that TBP decreases the Antp transcriptional activity in a similar manner as previously described with TFIIEβ, Exd and BIP2 [13, 22]. These results are in accordance with Hox interactions that also decrease transactivation activity in presence of TBP [15–18]. In addition, the PolyQ mutants of Antp were not significantly affected by the presence of TBP, thus confirming the relevance of PolyQ in the Antp-TBP interaction. The PolyQ region of Fushi Tarazu interacts with TFIIB and the PolyQ region of AbdA acts as transcriptional activation domain in the regulation of *decapentaplegic* and *wingless* [44–46] indicating that they are relevant for the interaction with the PIC for transcriptional regulation.

The PolyQ stretch of TBP is involved in the interaction with several TFs [31, 47, 48], leading repression and enhancing transcription [15–18, 49–51] and it has been proposed that it acts like a “communication port” for interaction with other TFs near the promoter in transcriptional regulation [52–55].

To our best knowledge, we describe for the first time the formation of trimeric complexes between Antp-TBP and TFIIEβ or Exd using a combined BiFC-FRET assay.

In order to validate the new Antp-TBP-TFIIEβ complex formation, we tested Antp mutations that disrupt protein–protein interactions and decrease FRET values. Previous reports both in vitro and in vivo show that Antp-TFIIEβ interaction is HD-dependent, specifically through HD residues 32 and 36 [14, 22, 56]. These results clearly corroborate the trimer formation in a similar way as described for Jun-Fos-p65 [26] and supports the importance of the PolyQ region as well as two single positions in the HD for the trimer formation. It was previously suggested that a trimeric complex can be formed by a protein that interacts with other proteins in an independent way acting as a bridge [23, 57, 58]. Given that BiFC assays have shown that TBP barely interacts with TFIIEβ (unpublished results), it seems reasonable to speculate that Antp mediates the trimeric complex acting as a bridge between TBP through its PolyQ region and TFIIEβ via its HD.

Interestingly, the Antp-TBP-TFIIEβ complex shows a highly significant increase of Antp transcriptional activity (238%). This activity is due to the presence of the trimer, given the decrease of the transactivation activity when only the dimers are present, and the disruption of the interaction with TBP and TFIIEβ by using Antp mutants. It is important to indicate that trimer-enhanced transactivation is dependent on protein–protein interaction with TFIIEβ, since Antp^I32A−H36A^ mutant decreased the trimer activity as previously described for its co-expression with Antp [22]. These results support the key role of these two single HD amino acids in the assembly of the trimeric complex and its transcriptional function. In vivo*,* Antp-TFIIEβ is required for antennae transformation into mesothoracic appendages, and this effect depends on residues 32 and 36 [22]. Our TFIIEβ trimer results in Antp transactivation could suggest an in vivo scenario in which Antp potentiates its transcriptional function in target genes.

We also corroborated that Exd forms a trimeric complex with Antp-TBP using Antp mutants that decreased FRET values significantly by disrupting Antp-Exd and Antp-TBP interactions, suggesting that the PolyQ regions and the YPWM motif are important mediators in this complex [13]. The Exd trimer decreased transcriptional activity, in contrast to the TFIIEβ trimer. This reduction is observed despite Exd binding to BS2 sites and partially activating LUC reporter compared to Antp (Fig. S4). In the same manner, other reports show that the trimer complex MEIS1-PBX-HOXA9 and the dimer PBX-HOXA9 did not transactivate a reporter gene with PBX-HOXA9 binding sites in myeloid leukemia [59]. On the other hand, Ubx-Exd-Hth can repress transcription, whilst Antp-Exd-Hth does not repress transcription in vivo [60].

When we analyzed whether Antp-TBP forms a trimer with BIP2, we did not find a positive FRET value compared to the Jun-Fos-p65 controls (Fig. S3). Additionally, we did not find significant differences between the Antp mutants used to disrupt its interaction with TBP and BIP2, when compared to Antp wild type.

Altogether, our results support that the trimeric complexes with TFIIEβ and Exd could modulate gene expression by activation or repression.

Our results raised the question of how the dimeric and trimeric interactions of Antp with GTFs and Exd contribute to transcriptional regulation within the Pol II Preinitiation Complex (PIC) at promoters and enhancers. When Antp is co-expressed with TBP, TFIIEβ, BIP2 or Exd, its transactivation activity is diminished [13, 22] hence we could speculate that Antp dimers are involved in repression transcriptional activities. Within the PIC, TBP recruits TFIIB by protein–protein interaction through the TBP PolyQ stretch [47]. Therefore, Antp interaction with TBP could inhibit transcription by a “squelching” effect, preventing the recruitment of TFIIB in the establishment of the PIC [61]. Similary, Eve acts as a repressor in *Drosophila* embryogenesis, interacting with TBP and blocking transcription in vitro by preventing TFIID-TATA box interaction [62]. Other HD proteins like Msx1 or Pax5 also interact with TBP, leading to transcriptional repression [15–18]. In the same manner, it has been determined that the zinc-finger TF Krüppel (Kr), a *Drosophila* segmentation protein for early embryonic development, interacts with TFIIEβ for transcription repression and this interaction is DNA-binding dependent [63].

The high level of transactivation activity of the Antp-TBP-TFIIEβ trimer indicates an activation scenario for transcription in which Antp may serve as a GTF recruiter to assemble or stabilize the PIC. This can be done by allowing TBP to bind DNA for initiation, and/or TFIIEβ to promote TFIIH activities for transcriptional elongation. There are a number of HD TFs that participate in protein–protein interactions with activation functions, for instance, Kr interacts with TFIIB [63] and Med19 interacts with Antp and other homeoproteins for Ubx target gene activation in *Drosophila* [19].

The Antp trimeric complex with TBP and TFIIEβ may operate as an anchor between promoters and enhancers at Antp target genes, potentiating transcription. Moreover, at the chromatin level, it has been established that Exd and Hth cooperate with Hox proteins in chromatin opening [64], and Ubx binds DNA to open and close chromatin and modulate transcription during *Drosophila* haltere development [65]. It would be of great interest to determine whether the Antp trimer complexes with TBP, TFIIEβ and Exd participate in similar activities at the chromatin level.

## Conclusions

In this paper we describe the direct interaction of Antp with TBP and the new trimeric complexes with TFIIEβ and Exd but not with BIP2 in living cells. We also found that the PolyQ region of both proteins are necessary for the Antp-TBP interaction and that other Antp interacting domains, like the HD and YPWM, are relevant for the formation of trimeric complexes. These trimeric complexes can modulate transcriptional regulation and open the possibility to further explore their function in PIC formation and at chromatin level throughout the *Drosophila* genome.

## Methods

### Plasmids constructs

For BiFC assays, Antp, AntpΔN, AntpΔHD, Antp^AAAA^, AntpΔpolyQ as well as and hTBP coding sequences were generated by PCR from pPAC plasmids [13, 66] and hTBPQ80 was amplified from pUASTattB-hTBPQ80 [67]. The coding sequences were restriction-cloned in frame with C- or N-terminal of Venus (VC and VN, respectively) using the *Age*I and *Xba*I restriction sites of pCS2VNm9 and pCS2VC155 vectors [68]. Antp mutants (AntpQ5, AntpQ6, and AntpQ9) and hTBPΔQ40 were generated by site-directed mutagenesis (Quickchange II XL kit, Stratagene, La Jolla, CA, USA). For BiFC-FRET assays, BIP2 (2–89), Exd (144–376) and TFIIEβ coding sequences were PCR amplified and restriction-cloned in pECFP-N1 expression vector (Clontech, Mountain View, CA, USA) using *Apal* and *Agel* (Table S1). For transactivation assays, we used the pNPAC-Antp, -AntpΔN, -AntpΔHD, -Antp^AAAA^, -Antp^132A−H36A^ and -AntpΔPolyQ plasmids previously obtained [13, 22, 66]. pNPAC-AntpQ6 and pNPAC-AntpQ9 were constructed by PCR and restriction-cloned in *NotI.* Oligonucleotides used for cloning and site-directed mutagenesis in the constructs are listed in Table S1. All plasmid constructs were verified by DNA sequencing before cell co-transfections.

### BiFC and transactivation assays in cell culture

HEK293 cells were maintained at 37 °C in 5% CO_2_ and cultured in Dulbecco’s modified Eagle’s medium (DMEM) supplemented with 10% FBS (Invitrogen, Carlsbad, CA. USA) and 1% penicillin–streptomycin (Sigma-Aldrich, Saint Louis, MI, USA). For transfections, we seeded 2 × 10^5^ HEK293 cells per well in 6-well plates with glass coverslips, cultured for 48 h and co-transfected with 6 µg of DNA. The transfections were carried out with polietilenimine (PEI) 40 kDa (Sigma-Aldrich, Saint Louis, MI, USA), using 1 µL of PEI 15 mM for each microgram of DNA transfected. Interactions by BiFC were performed by co-transfecting the VC- and VN- constructs along with the plasmid pCAG-mCherry (donated by Ataúlfo Martínez-Torres) as a control for transfection efficiency and the BiFC percentage calculation, as previously described [22]. The coverslips were visualized 48 h after transfection using Zeiss Axio Imager 2 (Carl Zeiss, Germany) microscope and four different fields of cells were acquired in the green and red channels using the same parameters with a 20X objective in three independent experiments. The quantification of green (BiFC) and red fluorescence (cherry, transfection efficiency) was performed in ImageJ by converting the RGB image to 8 bits and using the brightness and contrast tools to discriminate the positive BiFC signal from the background. Each fluorescent cell (red or green) was quantified using the cell counter plug-in. Interaction percentages were calculated by counting the number of green cells per 100 red cells [22]. Representative images were acquired using Zeiss Axio Imager 2 (Carl Zeiss, Germany) microscope. For the transactivation assays, we co-transfected HEK293 cells in 6-well plates as described above with pPAC, pGLH11 reporter and pcopia-βGal (used to normalize the luciferase activity) as previously described [13]. The luminescence was determined 48 h after transfection using the Dual-Luciferase Reporter Assay System Kit (Promega, Madison, WI, USA) according to the manufacturer’s instructions. Transfections assays were performed in three independent experiments by triplicate.

### BiFC-FRET assays

For the analysis of trimeric interaction, the BiFC-FRET assays were performed in HEK293 cells maintained under standard cell culture conditions. Cells were seeded on 6-well plates, 48 h later transfections were carried out using 1 µL of PEI (40 kDa) 15 mM (Sigma-Aldrich, Saint Louis, MI, USA) for each microgram of DNA. Trimeric interactions by BiFC-FRET were performed co-transfecting the VC-, VN- and EFCP- constructs. BiFC-FRET image acquisition was done 48 h after transfection in an Olympus BX61W1 microscope; 10 nm size photographs were collected in spectral mode (420–660 nm) using 10 nm of stepsize under the confocal parameters 600v, 1X gain, 0% offset, and 10% laser potency with 20X objective. The BiFC-FRET quantification (*E-*value) was performed using ImageJ and the FRETTY plug-in. This plug-in uses 2D deconvolution spectral unmixing by comparing the donor and acceptor images to measure the energy transfer between Venus and ECFP [69]. For all assays, three independent experiments were performed. pBiFC-bJun-VN173, pBiFC-bJun-YN173, pBiFC-bFos-VC155, pBiFC-bFos-YC155, pBiFC-bFosΔZIP-VC155, pFlag-p65-Cerulean and pFalg-p65Δ25-Cerulean expression vectors used for BiFC-FRET standardization were kindly provided by Hu Chang-Deng [26].

## Supplementary Information

**Additional file 1:** **Supplementary Figure 1.** Alignment of amino acid sequences in Antp mutants. Antp WT  sequence comparison with Antp mutants showed: AntpΔN lacking the N-terminal region (amino acids 1-269), AntpΔHD without the HD (aminio acids 245-363), AntpAAAA  in which the YPWM was substituted by alanines, AntpΔPolyQ with deletion of PolyQ regions (amino acids 66-136), AntpQ9 with deletion of the 9-polyQ stretch (amino acids 110-118), AntpQ5 in which the 5-polyQ stretch was mutagenized to alanines (amino acids 123-127), AntpQ6 with deletion of three glutamines (amino acids 150-152) and substitution of three glutamines to alanines (amino acids 153-155).

**Additional file 2:** **Supplementary Figure 2.** Alignment of amino acid sequences in hTBP and mutants. hTBP protein sequence compared to hTBP mutants: TBPΔQ40 lacking the Poly-glutamine region (amino acids 50-102), and hTBPQ80 has an extension of 80 glutamine residues (amino acids 50-142).

**Additional file 3:** **Supplementary Figure 3.** Trimeric interaction p65-Jun-Fos by BiFC-FRET by Fretty algorithm. (A) Schematic representation of p65 fused to Cerulean (donor), BiFC by Jun-Fos interaction (acceptor), and energy transfer due to p65-Jun-Fos trimeric complex (FRET). (B) p65-Jun-Fos form a trimeric complex (E=0.42±0.22). (C) Deletion of 25 aminoacids from p65 (p65Δ) decreased the trimeric complex formation (E=0.13±0.07). (D) Fos mutation (FosΔ) reduced the formation of the trimeric complex (E=0.14±0.04). Color bar represents FRET intensity (Fire mode); darker color indicates low trimeric interaction levels and lighter color indicates high trimeric interaction levels. FRET images were acquired using the Fretty algorithm. (E) The graph shows statistical analyses of three independent triplicates using a one-way ANOVA for mean comparison, significance is indicated as ***=*p* ≤ 0.001, **=*p* ≤ 0.01, *=*p* ≤ 0.05 and Error bars correspond to standard error (p ≤ 0.001). Scale bar, 20 µm.

**Additional file 4:** **Supplementary Figure 4.** Transcriptional factors activity on pGLH11. (A) Schematic representation of pGLH11 luciferase reporter (LUC) containing a minimal Hsp70 promoter and eleven tandem copies of BS2 Antp binding sites. (B) The graphic shows the percentage of transactivation activity mediated by Antp, hTBP, TFIIEβ, Exd, and BIP2. pPAC shows the levels of transcription of the empty expression vector. Statistical analysis of three independent triplicates was made using a one-way ANOVA and the post-hoc test Tukey for mean comparison. Error bars correspond to standard deviation (*p* <0.005).

**Additional file 5:** **Supplementary Table 1.** List of oligonucleotides sequences used for plasmid constructions. 

## Data Availability

Not applicable.

## Abbreviations

BiFC: Bimolecular Fluorescent Complementation
FRET: Fluorophore Resonance Energy Transfer
ECFP: Enhanced Cyan Fluorescent Protein
Antp: Antennapedia
TBP: TATA binding protein
VN: N-terminal region of Venus fluorescent protein
VC: C-terminal region of Venus fluorescent protein
