# Supplementary figures and images for: Trimeric complexes of Antp-TBP with TFIIEβ or Exd modulate transcriptional activity
Source: Hereditas. 2022 May 30;159:23. doi: 10.1186/s41065-022-00239-8 (PMC9150345; doi:10.1186/s41065-022-00239-8)

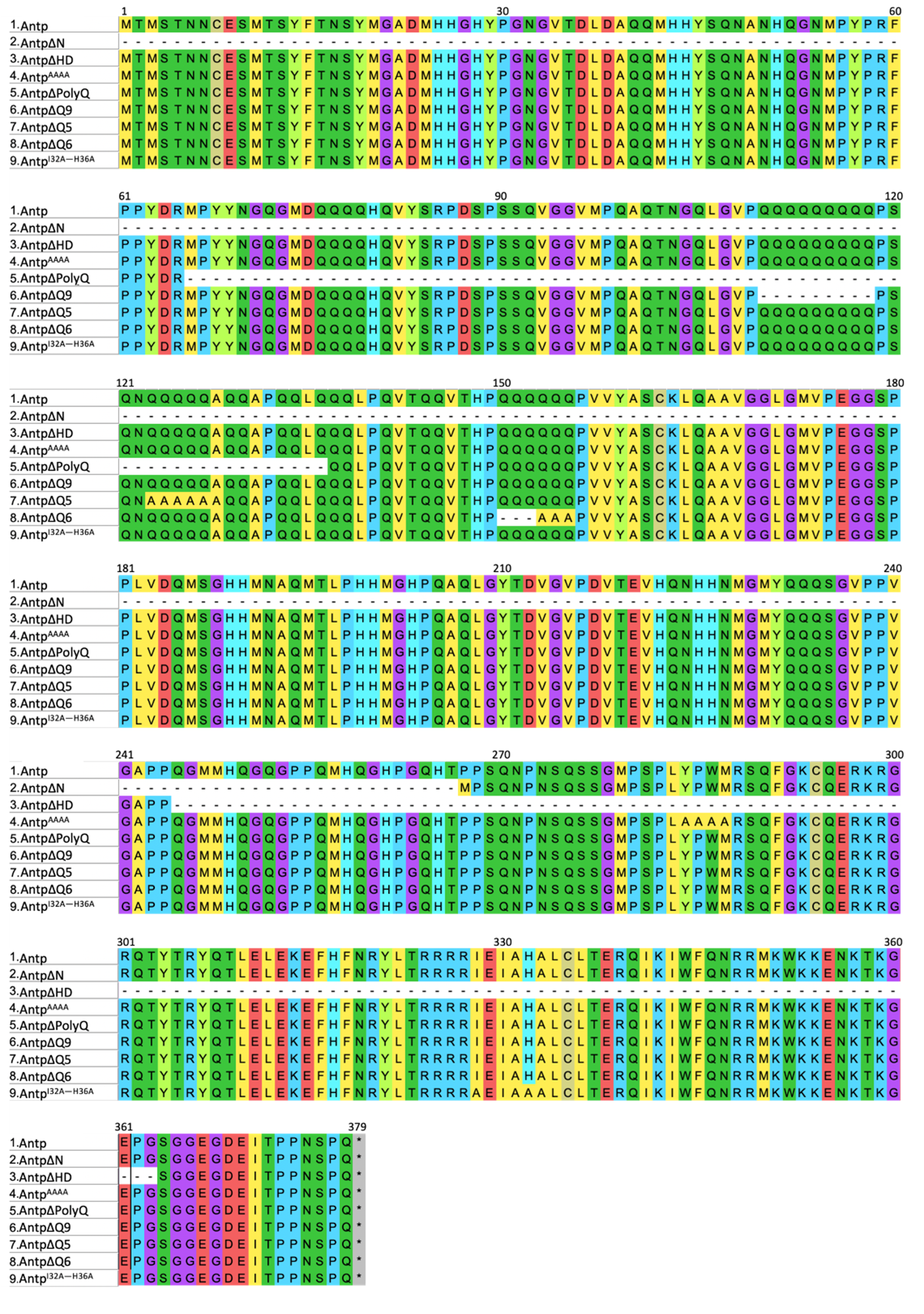

Supplement: Supplementary file 1 — Additional file 1: Supplementary Figure 1. Alignment of amino acid sequences in Antp mutants. Antp WT sequence comparison with Antp mutants showed: AntpΔN lacking the N-terminal region (amino acids 1-269), AntpΔHD without the HD (aminio acids 245-363), AntpAAAA in which the YPWM was substituted by alanines, AntpΔPolyQ with deletion of PolyQ regions (amino acids 66-136), AntpQ9 with deletion of the 9-polyQ stretch (amino acids 110-118), AntpQ5 in which the 5-polyQ stretch was mutagenized to alanines (amino acids 123-127), AntpQ6 with deletion of three glutamines (amino acids 150-152) and substitution of three glutamines to alanines (amino acids 153-155). [file 41065_2022_239_MOESM1_ESM.png]

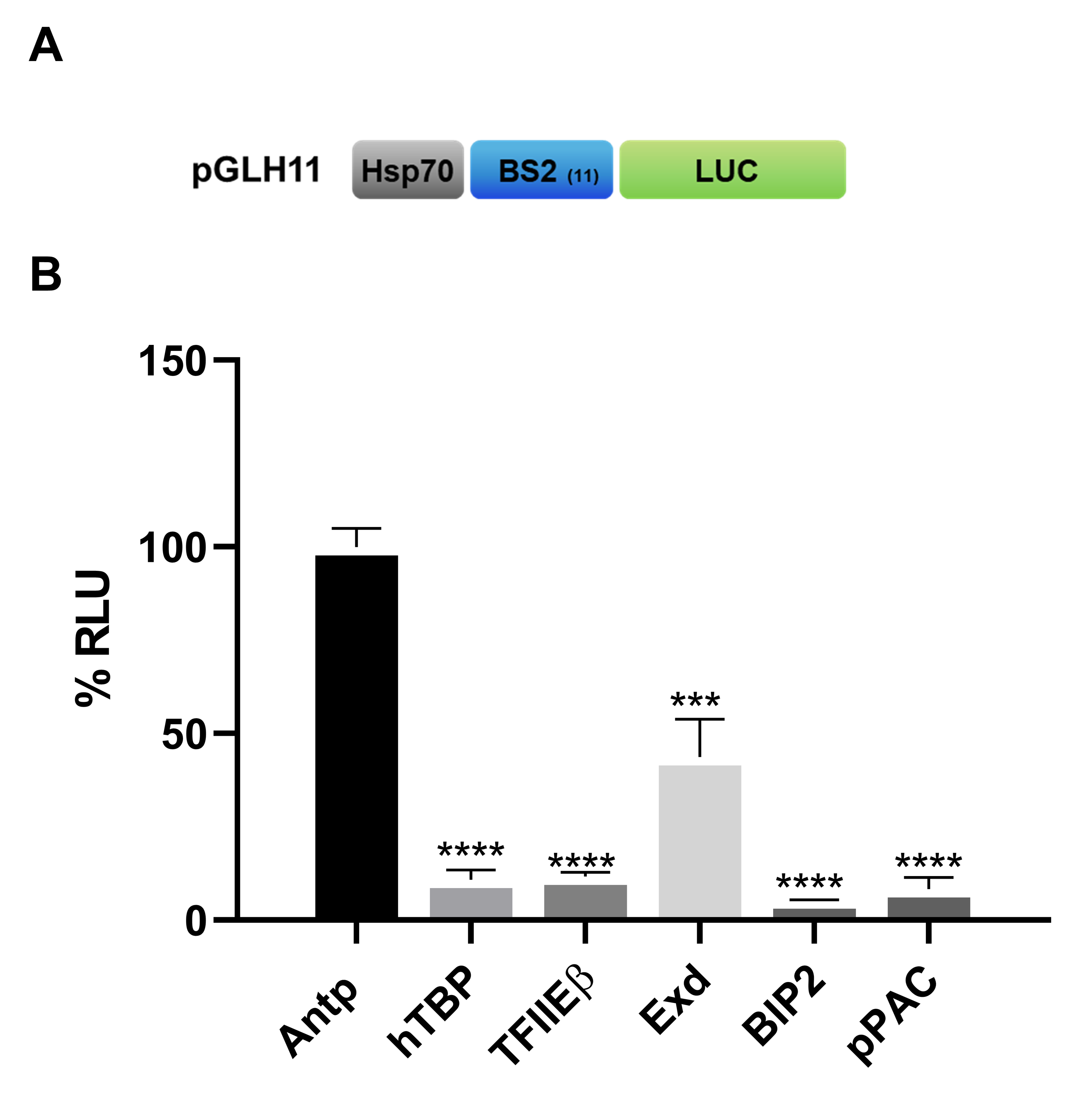

Supplement: Supplementary file 4 — Additional file 4: Supplementary Figure 4. Transcriptional factors activity on pGLH11. (A) Schematic representation of pGLH11 luciferase reporter (LUC) containing a minimal Hsp70 promoter and eleven tandem copies of BS2 Antp binding sites. (B) The graphic shows the percentage of transactivation activity mediated by Antp, hTBP, TFIIEβ, Exd, and BIP2. pPAC shows the levels of transcription of the empty expression vector. Statistical analysis of three independent triplicates was made using a one-way ANOVA and the post-hoc test Tukey for mean comparison. Error bars correspond to standard deviation (p <0.005). [file 41065_2022_239_MOESM4_ESM.png]
