# Supplementary material for: Trimeric complexes of Antp-TBP with TFIIEβ or Exd modulate transcriptional activity
Source: Hereditas. 2022 May 30;159:23. doi: 10.1186/s41065-022-00239-8 (PMC9150345; doi:10.1186/s41065-022-00239-8)
Supplement: Supplementary file 5 — Additional file 5: Supplementary Table 1. List of oligonucleotides sequences used for plasmid constructions. [file 41065_2022_239_MOESM5_ESM.pdf]

**Plasmid**	**Restriction enzymes**	**Oligonucleotide sequences**
pCS2VC155-Antp<br>pCS2VC155-AntpΔHD<br>pCS2VC155-Antp^{AAAA}<br>pCS2VC155-AntpΔ PolyQ	*Age*I / <u>*Spe*I</u>	Fwd5'- AAC*ACCGGT*ATGACGATGAGTACA<br>Rev3'-GCCT<u>*ACTAGT*</u>CGATCCTACTGCG
pCS2VC155-AntpΔN	*Age*I / <u>*Xba*I</u>	Fwd5'- AGATCTATG*ACCGGT*CCTTCCC<br>Rev3'- GCCT<u>*ACTAGT*</u>CGATCCTACTGCG
pCS2VC155-AntpQ9	Mutagenesis	Fwd5'- CCCTCGCAGAACCAGCAGC<br>Rev3'- AGGAACACCCAACTGACCG
pCS2VC155-AntpQ5	Mutagenesis	Fwd5'- CAGCGGCGGCGCAGCAGGCCCCACAGCAACTGC<br>Rev3'- CCGCCGCGTTCTGCGAGGGCTGTTGCTGCTG
pCS2VC155-AntpQ6	Mutagenesis	Fwd5'- GCAGCGGCGCCCGTCGTCTACGCCAGCTGC<br>Rev3'- TGCCGCCGCCGGATGTGTCACCTGTTGCGTCACC
pNPAC-AntpQ9<br>pNPAC-AntpQ6	<u>*Not*I</u>	Fwd5'- <u>*GCGGCCGC*</u>ATGACGATGAGTACAAAC<br>Rev3'- <u>*GCGGCCGC*</u>CGATCCTACTGCG
pCS2VNm9-hTBPΔQ40	Mutagenesis	Fwd5'- GTTCAGCAGTCAACGTCCCAG<br>Rev3'- AGACAGACTATTGGTGTTCTGAATAGGCTG
pCS2VNm9-TBP and<br>pCS2VNm9-TBPQ80	*Age*I / <u>*Xba*I</u>	Fwd5'- CGT*ACCGGT*ATGGATCAGAACAACAGCC<br>Rev3'- ACGT<u>*TCTAGA*</u>TTACGTCGTCTTCCTGA
pECFP-N1-TFIIEβ	*Apa*I-*Sma*I /<br><u>*Age*I</u>	Fwd5'- AT*GGGCCCGGG*ATCCATCCAAGCCTGTTGAGA<br>Rev3'- GC<u>*ACCGGT*</u>GCTCCACCTGATTTGCTGGAAGTA<br>ATGTCAGAGTAATC
pECFP-N1-Exd	*Apa*I-*Sma*I /<br><u>*Age*I</u>	Fwd5'- AT*GGGCCCGGG*ATGGGTGCTGACAATGCCATTGA<br>Rev3'- GC<u>*ACCGGT*</u>GCTCCACCTGAGGGGCTTAGATCCTG<br>ATGGAGATTTGG
pECFP-N1-BIP2	*Apa*I-*Sma*I /<br><u>*Age*I</u>	Fwd5'- AT*GGGCCCGGG*ATGATGGCAGACAGATACGCT<br>Rev3'- GC<u>*ACCGGT*</u>GCTCCACCTGAAGGCTCGACATTA<br>CCTATGTAGTC
